# Nutraceuticals as therapeutic agents for atherosclerosis

**DOI:** 10.1016/j.bbadis.2018.02.006

**Published:** 2018-05

**Authors:** Joe W.E. Moss, Jessica O. Williams, Dipak P. Ramji

**Affiliations:** Cardiff School of Biosciences, Cardiff University, Sir Martin Evans Building, Museum Avenue, Cardiff CF10 3AX, UK

**Keywords:** ANCHOR, Effect of AMR101 (Icosapent Ethyl) on Triglyceride Levels in Patients on Statins With High Triglyceride Levels (≥200 and <500 mg/dL), Apo, apolipoprotein, ASAP, Antioxidant Supplementation in Atherosclerosis Prevention, CANTOS, Canakinumab Anti-inflammatory Thrombosis Outcomes Study, CHD, coronary heart disease, CIRT, Cardiovascular Inflammation Reduction Trial, CoCoA, Cocoa, Cognition and Aging, CoQ_10_, coenzyme Q_10_, CRP, C-reactive protein, CVD, cardiovascular disease, DGLA, dihomo-gamma-linolenic acid, ECM, extracellular matrix, EPA, eicosapentaenoic acid, ESRD, end stage renal disease, EUROLIVE, Effect of Olive Oils on Oxidative Damage in European Populations, FMD, flow-mediated dilation, FOURIER, Further Cardiovascular Outcomes Research With PCSK9 Inhibition in Subjects With Elevated Risk, GISSI, Gruppo Italiano per lo Studio della Sopravvivenza nell'infarlo, GLA, gamma-linolenic acid, GPR, G-protein-coupled receptor, HDL, high-density lipoprotein, IL, interleukin, IMPROVE-IT, Improved Reduction of Outcomes: Vytorin Efficacy International Trial, JELIS, Japan EPA Lipid Intervention Study, KIHD, Kuopio Ischaemic Heart Disease Risk Factor, LDL, low-density lipoprotein, LDL-C, LDL-cholesterol, LDLR, LDL receptor, MAGMA, Multi-Analyte, Genetic, and Thrombogenic Markers of Atherosclerosis, MI, myocardial infarction, MARINE, Multi-center, plAcebo-controlled, Randomized, double-blINd, 12-week study with an open-label Extension, MRS-ROZE, Multicenter Randomized Study of ROsuvastatin and eZEtimibe, NO, nitric oxide, OMEGA, Effect of Omega 3-Fatty Acids on the Reduction of Sudden Cardiac Death After Myocardial Infarction, OPTILIP, The Quantification of the Optimal n-6/n-3 ratio in the UK Diet, ORIGIN, Outcome Reduction with an Initial Glargine Intervention, oxLDL, oxidized LDL, PCSK9, proprotein convertase subtilisin/kexin type 9, PRECISE-IVUS, Plaque Regression With Cholesterol Absorption Inhibitor or Synthesis Inhibitor Evaluated by Intravascular Ultrasound, PREDIMED, Prevención con Dieta Mediterránea, PUFA, polyunsaturated fatty acid, PWV, pulse wave velocity, REDUCE-IT, Reduction of Cardiovascular Events with EPA – Intervention Trial, STRENGTH, STatin Residual Risk Reduction With EpaNova in HiGh CV Risk PatienTs With Hypertriglyceridemia, SU.VI.MAX, Supplementation en Vitamines et Mineraux Antioxydant, TG, triacylglycerol, TMAO, trimethylamine-*N*-oxide, VEAPS, Vitamin E Atherosclerosis Prevention Study, VLDL, very low-density lipoprotein, Atherosclerosis, Cardiovascular disease, Nutraceuticals, Polyunsaturated fatty acids, Polyphenols

## Abstract

Atherosclerosis, a chronic inflammatory disorder of medium and large arteries and an underlying cause of cardiovascular disease (CVD), is responsible for a third of all global deaths. Current treatments for CVD, such as optimized statin therapy, are associated with considerable residual risk and several side effects in some patients. The outcome of research on the identification of alternative pharmaceutical agents for the treatment of CVD has been relatively disappointing with many promising leads failing at the clinical level. Nutraceuticals, products from food sources with health benefits beyond their nutritional value, represent promising agents in the prevention of CVD or as an add-on therapy with current treatments. This review will highlight the potential of several nutraceuticals, including polyunsaturated fatty acids, flavonoids and other polyphenols, as anti-CVD therapies based on clinical and pre-clinical mechanism-based studies.

## Highlights

• Atherosclerosis, an inflammatory disorder of the vasculature, is a major underlying cause of cardiovascular disease.
• Current therapies are not fully effective and many promising pharmaceutical leads have failed at the clinical level.
• Nutraceuticals are emerging as promising alternatives for prevention of atherosclerosis or as add-on with current therapies.
• Unfortunately, research on nutraceuticals has considerably lagged those on pharmaceuticals.
• This review summarizes our current knowledge on key nutraceuticals from clinical and pre-clinical mechanism-based studies.

## 1 Introduction

Cardiovascular disease (CVD) accounts for about 31.5% of all global deaths [1]. An estimated 92.1 million US adults have CVD and it is expected that this will increase to 43.9% of the adult population by 2030 due to a rise in obesity and diabetes, thereby imposing a greater burden on the healthcare services [1].

Atherosclerosis, an inflammatory disorder of the vasculature, is the major underlying cause of CVD [2]. Atherosclerosis is initiated by endothelial dysfunction predominantly due to the accumulation of apolipoprotein (Apo)B containing lipoproteins, particularly low-density lipoprotein (LDL) [2]. Endothelial cell dysfunction leads to the infiltration of LDL particles and their subsequent oxidation to oxidized LDL (oxLDL). In addition, there is an increase in both the secretion of chemokines from the cells and the expression of adhesion proteins on their surface that triggers the recruitment of immune cells, particularly monocytes [2]. The monocytes then differentiate into macrophages, which take up modified LDL to transform into foam cells [2,3]. Over time, foam cells undergo apoptosis and necrosis and this coupled with their defective clearance (efferocytosis) leads to the formation of a lipid-rich necrotic core associated with a chronic inflammatory response mediated by a range of cytokines [4,5]. The lipid-rich core is covered by a fibrous cap formed by the extracellular matrix (ECM) produced by smooth muscle cells that proliferate and migrate from the media into the intima [2]. The fibrous cap provides lesion stability and excessive degradation of the ECM due to increased expression and activity of a range of proteases in response to inflammatory mediators causes plaque rupture, subsequent thrombotic events and clinical complications of atherosclerosis [3].

Although the different stages in the pathogenesis of atherosclerosis are all promising therapeutic targets, major success has emerged from agents that control lipid homeostasis. Statins that lower LDL-cholesterol (LDL-C) and have additional pleiotropic effects are widely used [6]. However, there is a considerable residual risk of CVD in patients on statin therapy with some individuals unable to achieve target LDL-C goals even with high doses or are intolerant to the drug [6]. High dose statin therapy is also occasionally associated with side effects such as non-allergic rhinitis, rhabdomyolysis and hyperglycemia though some of these are debatable [7]. Substantial research has therefore been carried out on alternative therapies with some recent successes. For example, ezetimibe, an inhibitor of intestinal Niemann-Pick C1-like protein, which is involved in the uptake of dietary cholesterol, has shown some promise in lowering plasma LDL-C and in improving cardiovascular outcomes as demonstrated by some recent trials: Improved Reduction of Outcomes: Vytorin Efficacy International Trial (IMPROVE-IT) [8]; Plaque Regression With Cholesterol Absorption Inhibitor or Synthesis Inhibitor Evaluated by Intravascular Ultrasound (PRECISE-IVUS) [9]; and Multicenter Randomized Study of ROsuvastatin and eZEtimibe (MRS-ROZE) [10]. Inhibition of proprotein convertase subtilisin/kexin type 9 (PCSK9), which binds to the LDL receptor (LDLR) and targets it for lysosomal degradation, is also effective in reducing LDL-C and cardiovascular events [11]. Several Phase III clinical trials were initiated on three PCSK9 antibodies, evolocumab, alirocumab and bococizumab [11]. The outcome of the Further Cardiovascular Outcomes Research With PCSK9 Inhibition in Subjects With Elevated Risk (FOURIER) trial on evolocumab involving 27,564 patients demonstrated CVD benefit from lowering of LDL-C levels below current targets [12]. On the other hand, the sponsors stopped the trials on bococizumab early due to high rates of antidrug antibodies [13]. Nevertheless, significant benefits were seen in high-risk patients [14]. Other avenues for inhibiting PCSK9 actions are also being pursued; for example, sustained reduction of LDL-C has recently been shown for Inclisiran, a long-acting synthetic small-interfering RNA that selectively targets PCSK9 [15].

The successes detailed above have been outnumbered by disappointing outcomes on many promising pharmaceutical agents [16]. Despite the extensive evidence associating increased plasma high-density lipoprotein (HDL) levels with a reduced risk of CVD, clinical trials using orally active, HDL raising agents have largely failed [17]. Clinical trials on inhibitors of cholesteryl ester transfer protein have also been disappointing [17]. Due to atherosclerosis being an inflammatory disorder, approaches that dampen inflammation are also being pursued [4]. Promising outcomes were recently obtained with the Canakinumab Anti-inflammatory Thrombosis Outcomes Study (CANTOS) that evaluated the potential of attenuating inflammation using a neutralizing interleukin (IL)-1β antibody, in order to reduce cardiovascular events in patients with prior myocardial infarction (MI) [18]. Similarly, the Cardiovascular Inflammation Reduction Trial (CIRT) is investigating the anti-inflammatory potential of low dose methotrexate [4]. It should however be noted that because of the risks associated with manipulating systemic inflammation (e.g. predisposition to infections), such approaches will have to be restricted to high risk patients until effective technologies to target them to the atherosclerotic lesions are developed. Such issues together with the disappointing outcomes on numerous pharmaceuticals detailed above, and the anticipated future increase in CVD-burden due to global raise in risk factors such as obesity and diabetes, highlight the urgent need for more research on alternative agents in the prevention and treatment of atherosclerosis.

Diets rich in fruit, vegetables, fish, cereal grains or olive oil have all been associated with cardiovascular health benefits [19]. Nutraceuticals, products derived from food sources that have health benefits beyond their nutritional value, have recently generated substantial interest in the prevention of atherosclerosis and as an add-on to current therapies for several reasons. Because nutraceuticals are derived from food sources, they generally do not suffer from the same issues of tolerability and safety that are often associated with pharmaceuticals especially given the long-term use required for the management of risk factors for CVD. In addition, nutraceuticals often have pleotropic effects with inhibitory actions on multiple pro-atherogenic changes thereby making them the agents of choice in conjunction with pharmaceutical drugs or other lifestyle changes. Nutraceuticals would be particularly useful for individuals that either have borderline risk factors for CVD, where there is likely to be particular reluctance to take pharmaceuticals due to various side effects, or are intolerant to drugs (e.g. statins) or have other issues such as sarcopenia. Furthermore, nutraceuticals may be more cost-effective add-on therapies for the management of dyslipidemia and other CVD risk factors for many individuals on current therapies (e.g. statins and/or ezetimibe) compared to more powerful but expensive alternatives that are in development (e.g. antibodies against PCSK9 and other proteins).

**Fig. 1:**
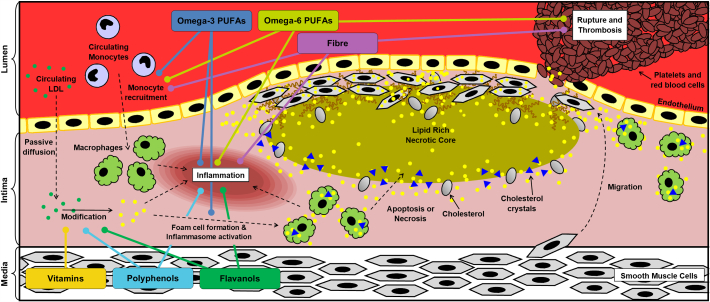
The actions of nutraceuticals on different stages in the pathogenesis of atherosclerosis. The different steps in the pathogenesis of atherosclerosis (modification of LDL, recruitment of immune cells such as monocytes, their differentiation into macrophages and subsequent transformation to foam cells, apoptosis and necrosis of such foam cells to form lipid-rich necrotic core, inflammasome activation by cholesterol crystals and other factors, stabilization of plaques by extracellular matrix produced by vascular smooth muscle cells and plaque rupture) are shown together with points where some key nutraceuticals mediate their actions. See text for more details.

Unfortunately, despite the promise detailed above, nutraceutical research has considerably lagged that on pharmaceutical agents on two key aspects, deeper mechanistic insight and large clinical trials. This is also reflected by the general paucity of published literature on nutraceuticals in the cardiovascular field compared to that on pharmaceuticals. The purpose of this review is to highlight some of the most promising nutraceuticals that are emerging as potential treatments and preventatives of atherosclerosis disease development. We discuss the effects of these nutraceuticals on clinical outcomes and provide both pre-clinical and mechanism-based studies where appropriate. Due to space limitations, we have been unable to address the outcomes of all published literature on these nutraceuticals. Nevertheless, we have tried to provide a balanced coverage taking both positive and negative evidence into account together with possible directions that should form the focus of future studies. Key outcomes from human studies on the major nutraceuticals addressed in this review are shown in Table 1. Some of the discrepancies seen in the literature reflect differences in the size and composition of the participants (e.g. mix of healthy, obese and patients with high risk for CVD), dose of the agent and duration of the intervention. Overall, epidemiological studies only indicate associations that are not necessarily causal with cohort studies being more informative and clinical trials providing the highest level of evidence. Considerations of the mechanisms of action of dietary components add to the plausibility of the results from the various studies. Fig. 1 therefore provides a summary of the steps in the pathogenesis of atherosclerosis where some of the nutraceuticals exert their actions and these are dealt in more detail in the relevant section of the review. In general, a given nutraceutical dampens multiple pro-atherogenic events rather than drastically inhibiting one or a few as is the case with pharmaceutical agents. There are of course many other nutraceuticals that have been less well studied and are therefore not addressed here in detail because of space constraints. Table 2 lists some of these other emerging nutraceuticals with a brief summary of their actions.

**Table 1:** Summary of major findings from human studies.

Nutraceutical	Trial name/first author	Number of participants	Study type	Major findings	Ref
Vitamins	Physicians' Health Study II	14,641	CL	No positive effects of Vitamins C or E on any major CVD outcomes Vitamin E increased risk of stroke	21
	SU.VI.MAX subset	1162	CL	Vitamin C and E increased number of plaques and decreased pulse wave velocity	22
	VEAPS	353	CL	No cardiovascular protective effects observed following vitamin E supplementation	23
	MRC/BHF Heart Protection Study	20,536	CL	No benefit of Vitamin C, E and beta-carotene supplementation	24
	GISSI-Prevenzione	11,324	CL	No benefit of vitamin E	25
	ASAP	520	CL	Vitamin C and E reduced carotid artery atherosclerosis in smoking men	26
	Woman's Health Study	39,876	CL	24% reduction in CVD mortality in women receiving vitamin E	27
	Karppi et al.	840	EP	High serum carotenes is protective in early atherosclerosis	29
Omega-3	GISSI-Prevenzione	11,324	CL	15% reduction in total number of deaths and non-fatal CVD-related events 45% reduction in sudden cardiac deaths	25
	JELIS	18,645	CL	19% reduction in major CVD-related events Most effective as a secondary preventative	33
	MAGMA	600	CL	Lowered TG, VLDL and oxLDL levels No additional benefit to those already taking lipid lowering therapies	35
	OMEGA	3851	CL	No cardiovascular protective effects observed after 1-year follow-up	36
	ORIGIN	12,536	CL	No cardiovascular protective effects observed after 6-years follow-up	37
	MARINE	229	CL	Lowered TG levels	38
	ANCHOR	702	CL	Lowered TG, non-HDL, LDL and total cholesterol levels in statin receiving patients	39
	REDUCE-IT	8000	CL	To be completed in 2018	41
	STRENGTH	13,000	CL	To be completed in 2019	42
Omega-6	OPTILIP	258	CL	Optimization of Omega 6:3 Ratio to 3:1 reduces TG and LDL levels	54
	Katan et al.	340,000	EP	Replacement of saturated fats with PUFA reduces CVD risk	49
	Oh et al.	78,778	EP	Inverse relationship between PUFA and CVD in women with BMI over 25	50
	Laaksonen et al.	1551	EP	Linoleic acid is cardio protective and reduces overall mortality	51
	Tomiyama et al.	2206	EP	Serum CRP levels correlate with increased omega-6 serum levels.	53
Polyphenols	EUROLIVE	200	CL	Correlation between polyphenol intake and plasma HDL levels Reduced TG and oxidative stress markers	64
	Tjelle et al.	134	CL	Reduced blood pressure	60
	Shema-Didi et al.	101	CL	Reduced blood pressure and improved lipid profile in hemodialysis patients	61
	Panahi et al.	117	CL	Improved inflammatory and oxidative condition of metabolic syndrome patients	65
	Ras et al.	70	CL	No change in blood pressure	62
	Bondia-Pons et al.	78	CL	No change in blood pressure	63
	PREDIMED	7172	CH	Increased polyphenol intake resulted in a reduced mortality risk	70
	PREDIMED subset	200	CH	Polyphenol intake reduced blood pressure and increased plasma NO levels	71
	PREDIMED subset	573	CH	Reduced blood pressure in elderly individuals Decreased TG levels but no changes in total cholesterol, HDL or LDL levels	72
	PREDIMED subset	1139	CH	Reduced plasma levels of several inflammatory biomarkers	73
Flavonoids	CoCoA	90	CL	Reduced blood pressure and lipid peroxidation	90
	Flaviola Health study	100	CL	Increased FMD and plasma HDL levels Decreased blood pressure, arterial stiffness, total and LDL cholesterol levels	91
	PREDIMED	7172	CH	Increased flavanol intake correlated with reduced CVD risk	69
	Rassaf et al.	57	CL	Improved FMD and reduced blood pressure	84
	The Zutphen Elderly Study	805	CH	Reduced mortality from CHD and incidence of MI	79
	The Caerphilly Study	1900	CH	No change in incidence of ischemic heart disease	81
Fiber	Ramos et al.	116	CL	No additional benefit when combined with other lipid lowering therapies	96
	Merchant et al.	46,032	EP	Increased fiber intake associated with reduced peripheral arterial disease risk	93
	Oh et al.	78,779	EP	Increased fiber intake reduced the risk of stroke in women	94
	Liu et al.	39,876	EP	Increased fiber consumption was correlated with reduced CVD risk	95

See text for further details on these studies, including dosage, trial duration, intervention type and the type of populations. CH, cohort study; CL, clinical trial; EP, epidemiological study.

**Table 2:** Other emerging nutraceuticals and their potential benefits.

Nutraceutical	Summary of findings	Ref
Allicin	Attenuated atherosclerosis in mouse model systems. Garlic supplementation in human trials produced short-term reduction in plasma levels of LDL, total cholesterol and TG	113–114
Berberine	Reduced plaque size, inflammation and oxidative stress in ApoE deficient mice. Decreased plasma levels of total cholesterol and LDL in human studies	115–119
Butyrate	Reduced atherosclerosis and oxidative stress in ApoE deficient mice	101, 102
Carnosine	No changes in serum cholesterol and CRP levels or blood pressure in humans. Reduced vascular inflammation and foam cell formation, as well as a decrease in serum TG levels in ApoE deficient mice	121–124
Coenzyme Q_10_	Attenuated atherosclerosis in ApoE deficient mice and promoted cholesterol efflux and endothelial function in human subjects	127–129
Curcumin	Multiple anti-atherogenic actions in mouse model systems and decreased C-reactive protein levels in human studies	66–68
Lycopene	Reduced levels of LDL and pro-inflammatory cytokines in human subjects	125, 126
Phytosterols	Attenuated atherosclerosis in mouse model systems and reduced plasma LDL in human subjects	130–134, 136–137

## 2 Antioxidant vitamins: great promise but major disappointments

The oxidation of LDL and the production of free radicals within the endothelium is a key early pro-atherogenic event and might also play important roles at later stages of the disease [2,3]. Appropriate dietary modifications or supplementation with agents that prevent LDL oxidation may therefore aid in managing the risk of CVD. Throughout the 1990s, antioxidant vitamins were at the forefront of research into the prevention of CVD. The beneficial effects of three main types of antioxidant vitamins; beta-carotene, Vitamin C and Vitamin E, have been investigated in numerous animal studies and human trials. These are essential vitamins as they cannot be produced or stored by the body and therefore must be acquired in the diet from fruit and vegetables or from supplements [20].

Pre-clinical studies on all antioxidant vitamins showed great promise in demonstrating an inverse relationship with CVD risk factors/events [20]. However, results from clinical trials have been less consistent and somewhat disappointing [20]. A large, randomized controlled Physicians Health Study II, involving 14,641 men, investigated the role of vitamins C and E independently and as a multivitamin on a number of CVD outcomes over a period of 8 years [21]. The study found no positive effects on CVD outcomes in any of the treatment groups in comparison to the placebo control. In fact, Vitamin E supplementation caused increased risk of haemorrhage and stroke [21]. The outcome of the Supplementation en Vitamines et Mineraux Antioxydant (SU.VI.MAX) subset trial involving 1162 men and women also supports the lack of cardiovascular protective effects of antioxidant supplementation [22]. The percentage of subjects with carotid plaques was higher following intervention despite reduced arterial stiffness as indicated by pulse wave velocity (PWV) [22]. Other studies, such as the Vitamin E Atherosclerosis Prevention Study (VEAPS) showed that in low risk individuals, vitamin E supplementation had no impact on CVD outcomes over a 3-year follow-up period despite reduction in LDL oxidation [23]. In addition, no significant reductions in 5-year mortality from incidence of any type of vascular disease were found in a large randomized, placebo-controlled trial of 20,536 adults (aged 40–80 years) with coronary disease, other occlusive arterial disease or diabetes involving antioxidant vitamin supplementation (600 mg vitamin E, 250 mg vitamin C and 20 mg beta-carotene daily) [24]. Similarly, no benefit of vitamin E was seen in the GISSI (Gruppo Italiano per lo Studio Sopravvivenza nell'infarto)-Prevenzione trial [25] (see Section 3 for more details on this trial). In contrast, the Antioxidant Supplementation in Atherosclerosis Prevention (ASAP) trial involving 520 men and postmenopausal women showed some positive outcomes after receiving both vitamin C and E supplementation for 3 years [26]. The progression of common carotid atherosclerosis was retarded in men who were regular smokers. In addition, the authors suggested that this could be generalizable to all men and did not rule out a small benefit to women [26]. This latter proposition has some support from the Woman's Health Study on vitamin E supplementation involving 39,876 healthy US women [27]. In the 10-year follow-up period, there was a significant 24% reduction in cardiovascular death in the supplement group [27]. Inverse correlation was also found between carotenoid antioxidant supplementation and common carotid artery intima-media thickness in 1212 elderly Finnish men [28]. More recently, in 840 middle-aged men from Eastern Finland, high serum carotenoid concentration was found to be protective against early atherosclerosis [29].

Despite a large number of trials that have taken place over the last 20 years, conflicting outcomes make it difficult to determine any beneficial effects of antioxidant therapies. A major limitation is differences in the selection criteria of participants for these trials such as gender, existing risk factors for CVD and ethnicity. These aspects need to be taken into consideration in future trials to delineate the potential of antioxidant vitamins in the prevention of CVD.

## 3 Omega-3 polyunsaturated fatty acids

During normal physiological conditions, the synthesis of eicosanoids, inflammatory response intermediaries, as well as the regulation of blood pressure and clotting can be influenced by polyunsaturated fatty acids (PUFAs) [30]. Dietary intake of some PUFAs is essential because they cannot be synthesized in vivo. Omega-3 PUFAs are regarded as anti-inflammatory and exert their effects via multiple mechanisms, including disruption of lipid rafts, altered cell membrane phospholipid composition, modulation of activation of key transcription factors and signaling pathways, and the ability to bind to G protein-coupled receptor (GPR) 120 [30]. Indeed, omega-3 PUFAs ameliorate atherosclerosis and even cause plaque regression in mouse model systems via several mechanisms such as lowering plasma cholesterol levels, alteration of monocyte subsets and their recruitment to lesions, and modulation of dendritic cell phenotype [31,32].

Over the last 20 years, two large trials, GISSI-Prevenzione and Japan Eicosapentaenoic acid Lipid Intervention Study (JELIS), have investigated the potential cardiovascular protective effects of omega-3 PUFAs [25,33]. During the GISSI-Prevenzione trial, 11,324 patients who had suffered a recent MI were randomized between 4 treatment groups; vitamin E, omega-3 PUFA supplementation, both or control [25]. At the 3.5-year follow-up, there was a significant 15% reduction in the number of deaths and non-fatal CVD-related events between the omega-3 receiving group and the control group. In addition, there was a 45% reduction in sudden cardiac deaths [25]. The JELIS trial involved 18,645 hypercholesterolaemic participants who received statins or eicosapentaenoic acid (EPA; an omega-3 PUFA) in combination with statins [33]. In patients with history of coronary artery disease (secondary prevention group), a significant 19% reduction in the number of major coronary events was observed in the participants taking EPA compared to the statin only group after an average 4.6-year follow-up. In patients with no history of coronary artery disease (primary prevention group), EPA reduced major coronary events by 18% though this was not significant [33]. Although these trials have shown omega-3 PUFAs to potentially exert cardiovascular protective effects both alone and in combination with statins, it is important to note that these were performed in a single country before current omega-3 dietary guidelines were introduced [34]. Consequently, any differences between countries (i.e. diet) can affect the outcomes and therefore they should be seen to provide encouraging but not irrefutable evidence for the benefits of omega-3 PUFA consumption [34].

More recent trials, including Multi-Analyte, Genetic, and Thrombogenic Markers of Atherosclerosis (MAGMA) [35], Effect of Omega 3-Fatty Acids on the Reduction of Sudden Cardiac Death After Myocardial Infarction (OMEGA) [36] and Outcome Reduction with an Initial Glargine Intervention (ORIGIN) [37], have failed to find any additional cardiovascular benefits from taking omega-3 PUFAs in combination with traditional therapies. The MAGMA trial involved 600 participants who were randomly assigned to receive fish oil supplementation only or in combination with their lipid lowering therapy [35]. Within the total population, fish oil consumption reduced levels of triacylglycerol (TG), very low-density lipoprotein (VLDL) and oxLDL but not in those patients already receiving lipid-lowering therapy [35]. Both the OMEGA and ORIGIN studies used omega-3 ethyl esters intervention in 3851 and 12,536 high-risk patients respectively [36,37]. Unfortunately, neither study was able to observe any additional cardiovascular protective effects from omega-3 PUFA intervention. However, a major limitation of these studies was the low dose of omega-3 PUFAs used together with participants that had relatively normal levels of TG (a risk factor for CVD) [2,3,34].

The effect of omega-3 PUFAs in participants with elevated TG levels was assessed in two preliminary trials known as Multi-center, Placebo-controlled, Randomized, Double-blind, 12 week study with an Open-label Extension (MARINE) and Effect of AMR101 (Icosapent Ethyl) on Triglyceride Levels in Patients on Statins With High Triglyceride Levels (≥200 and <500 mg/dL) (ANCHOR) [38,39]. Omega-3 PUFAs in the form of EPA ethyl ester decreased TG levels together with other lipid parameters without increasing LDL levels [38,39]. The two trials also detected a reduction in several markers of inflammation [40]. These findings formed part of the rationale to perform a large international trial known as Reduction of Cardiovascular Events with EPA – Intervention Trial (REDUCE-IT) [34,41]. The trial is expected to be completed in 2018 and has involved approximately 8000 hypertriglyceridemic participants spread over 11 countries. Participants have received icosapent ethyl (a purified EPA ethyl ester) in combination with statins for 6.5 years [34,41]. Another large ongoing international trial called STatin Residual Risk Reduction With EpaNova in HiGh CV Risk PatienTs With Hypertriglyceridemia (STRENGTH) on approximately 13,000 patients from 22 countries is assessing the effect of omega-3 carboxylic acids in combination with statins in participants with hypertriglyceridemia [42]. The trial is expected to be completed in 2019. It is hoped that these studies will provide more substantial evidence on whether omega-3 PUFA supplementation is capable of providing additional cardiovascular protective effects when taken in combination with statins.

## 4 Omega-6 polyunsaturated fatty acids

In terms of cardiovascular health, the roles of omega-3 and omega-6 PUFAs are generally considered antagonistic, with the former regarded as anti-inflammatory and the latter as pro-inflammatory. The recent increased consumption of omega-6 PUFA-rich vegetable oils has altered the ideal omega-6:omega-3 PUFA ratio from 4:1 to approximately 15:1, which has been linked to an increase in inflammatory disorders [43]. The American Heart Association therefore recommends consumption of at least 200 g of oily fish a week in order to reduce the omega-6:omega-3 PUFA ratio [44]. However, not all omega-6 PUFAs are pro-inflammatory with both gamma-linolenic acid (GLA) and dihomo-gamma-linolenic acid (DLGA) reducing inflammation [45] and acting in an anti-atherogenic manner in mouse model systems [46,47]. However, the published literature relating to omega-6 PUFAs and CVD in humans remains conflicting [48].

Pooled data from 11 cohort studies (340,000 individuals) showed that a reduction in the consumption of saturated fats replaced with a proportionally higher intake of omega-6 PUFAs significantly decreased coronary heart disease (CHD) [49]. This conclusion is also supported by several other studies in both men and women which have shown omega-6 PUFA levels to be inversely associated with CVD risk and death [50,51]. A prospective Nurses' Health Study on 78,778 women showed an inverse association between the intake of the omega-6 PUFA, linoleic acid, and the risk of CHD [50]. A prospective population-based study, Kuopio Ischaemic Heart Disease Risk Factor (KIHD), on 1551 middle-aged men over a 15-year follow-up also demonstrated substantial cardioprotective benefits of linoleic acid and a reduction in overall mortality [51]. A much smaller human study also showed a positive impact of GLA in decreasing levels of TG (by 48%) and increasing HDL (by 22%) [52]. However, not all human studies support a beneficial role for omega-6 PUFAs in the prevention of CVD. For example, a study of 2206 healthy Japanese men showed that high serum levels of omega-6 PUFAs was associated with elevated CRP, suggesting an unfavorable inflammatory profile [53]. Many studies have investigated the impact of manipulating the omega-3:omega-6 PUFA ratio rather than focusing solely on omega-6 PUFAs. Thus, the Quantification of the optimal n-6/n-3 ratio in the UK Diet (OPTILIP) study involving 258 participants revealed that an omega-6:omega-3 PUFA ratio of 5:1 and 3:1 was associated with a reduction in the levels of TG and small, dense LDL-C compared to a control diet of 10:1 ratio [54]. Overall, the various studies highlight the need for further human trials evaluating the role of omega-6 PUFAs on the prevention of CVD.

## 5 Polyphenols

Polyphenols occur naturally in plants and plant products, including fruits, nuts, vegetables, herbs, tea and coffee [43,55]. Examples of polyphenols include flavonoids present in tea, cocoa and blackberries, resveratrol in red wine, hydroxytyrosol in olives and curcumin in turmeric. Flavonoids have been the subject of increased recent research and are therefore addressed separately in the next section.

Due to their abundance in edible plants and established anti-oxidant and anti-inflammatory properties, there has been increased interest in the use of polyphenols to reduce the risk of CVD [43]. Early epidemiological studies were able to highlight the cardiovascular protective effects of polyphenols found in olives and grapes [43,55]. Since these early studies, hundreds of polyphenol compounds have been identified [43,55]. However the Mediterranean diet remains the gold standard for polyphenol dietary intake and therefore a large number of investigations have focused on the polyphenols commonly found within this diet (e.g. hydroxytyrosol) [43,55,56]. Indeed, the high concentration of polyphenols is often associated with the anti-inflammatory and anti-atherogenic actions of oils rich in monosaturated fatty acids such as olive oil and rapeseed oil [[57], [58], [59]].

A human study involving 134 hypertensive subjects showed a reduction in blood pressure following intake of polyphenol-enriched juices (250–300 mg/100 g) every day for 12 weeks when compared to those receiving the placebo control [60]. A reduction in blood pressure was also found in 101 hemodialysis patients, a population with a high risk for CVD, following treatment with pomegranate juice (0.7 mM polyphenols) three times a week for 1 year [61]. During this study, there was also a correlation between length of treatment time and improvements in HDL and TG levels [61]. Furthermore, the subset of participants who had pathologically high TG levels and low HDL levels at the start of study showed significant improvements in these factors, which were not observed in the placebo control [61]. However, other studies have failed to find an association between polyphenol intake and reduced blood pressure [62,63]. For example, a study involving 70 patients who were either pre- or stage 1 hypertensive was unable to find any changes in their blood pressure compared to the placebo control after 8 weeks of daily grape extract supplementation [62]. Another study on 78 obese patients who were randomized to receive one of the following diets for 8 weeks; low omega-3 PUFAs low polyphenol, high omega-3 PUFAs low polyphenol, low omega-3 PUFAs high polyphenol, or high omega-3 PUFAs high polyphenol, also found no changes in their blood pressure or total and LDL cholesterol levels [63]. Those on the high polyphenol diets did have reduced TG levels, however their HDL plasma concentrations were also reduced [63]. Upon further analysis of this study, there was an inverse correlation between LDL-C levels and the intake of a specific polyphenol, gallic acid. The use of gallic acid for the reduction of LDL-C levels therefore needs to be investigated further.

The Effect of Olive Oils on Oxidative Damage in European Populations (EUROLIVE) randomized clinical trial found a positive correlation between the concentration of polyphenols in olive oil and serum HDL levels in 200 healthy men following daily intervention for 3 weeks [64]. Furthermore, oxidative stress markers decreased with increased polyphenol concentrations and TG levels were reduced in all participants receiving olive oil [64]. The decrease in oxidative stress markers seen in the EUROLIVE trial has since been confirmed in a later study on 117 patients with metabolic syndrome [65]. Supplementation with polyphenols (1 g/day) in the form of curcuminoids for 8 weeks increased their superoxide dismutase levels while simultaneously lowering malondialdehyde plasma concentrations, indicating reduced oxidative stress [65]. In addition, those receiving curcumoids had reduced plasma CRP levels [65]. Consistent with this study, meta-analysis of clinical trials also revealed reduced CRP levels with curcumin though no effect was seen at the levels of plasma total cholesterol, HDL and LDL-C [66,67]. Indeed, many animal model studies have revealed anti-atherogenic actions of curcumin via multiple mechanisms (e.g. reduced oxidative stress and inflammation, regulation of cholesterol homeostasis) [68].

The Prevención con Dieta Mediterránea (PREDIMED) study is the largest trial investigating the potential cardiovascular protective effects of the Mediterranean diet [69,70]. The study involved 7172 Spanish patients with a high risk of suffering from a CVD-related event. These patients were randomized into three different diets; Mediterranean diet supplemented with extra virgin olive oil, Mediterranean diet supplemented with mixed nuts and a low-fat control diet [69,70]. Initial analysis of the study found that after approximately 5 years, those with the highest polyphenol intake had a 37% lower relative risk of all-cause mortality when compared to those with the lowest polyphenol intake [70]. Further in-depth analysis on a subset of 200 high-risk patients found that those on the Mediterranean diet had reduced blood pressure after 1 year compared to those on the control diet [71]. The analysis also found a positive association between total polyphenol intake and plasma nitric oxide (NO) levels, highlighting a possible mechanism by which polyphenols are able to improve endothelial function, resulting in vasodilation and a reduction in risk for CVD [71]. This improvement in blood pressure was also found in a subset of 573 elderly participants of the PREDIMED study after a 5-year follow-up [72]. These individuals also had reduced plasma TG and glucose concentrations, however there were no changes in their total cholesterol, HDL or LDL-C levels [72]. More recently, a substudy of 1139 high-risk participants revealed reduced plasma levels of several inflammatory biomarkers related to atherosclerosis with polyphenol intake [73]. Overall, the PREDIMED study shows an association between the Mediterranean diet and cardiovascular protective effects by improving vascular functions and reducing both blood pressure and inflammatory markers, rather than altering circulating lipoproteins. Further studies are required to determine the specific nutraceutical within the diet that provides the cardiovascular health benefits as the PREDIMED study provides supportive rather than conclusive evidence for the use of polyphenols for CVD. In addition, despite the potential benefits of the Mediterranean diet the PREDIMED study has uncovered, it still has its limitations; for example, all of the participants were recruited from a single country and therefore differences in lifestyle between countries may alter the outcomes of the study.

## 6 Flavonoids

Cardiovascular protective effects are frequently associated with a high intake of fruit and vegetables that contain flavonoids, which are therefore addressed in more detail [74]. Flavonoids have generated substantial interest because of their ability to inhibit both LDL oxidation [[75], [76], [77]] and platelet aggregation [78]. Although vast literature exists on anti-platelet activities of flavonoids via various signaling pathways, clinical trials have been low in numbers and produced inconsistent results (see [78] for a recent review). In relation to antioxidant activities, the Zutphen Elderly Study of 805 men aged 65–84 for a 5-year follow-up revealed an inverse correlation between flavonoid intake and mortality from CHD together with the incidence of MI [79]. Such correlation was also found following a 10-year follow-up [80]. However, a study of 1900 Welsh men aged 45–59 years, who were followed for 14 years, failed to reveal any protection from antioxidant flavonols, mainly from tea to which milk was added, and the incidence of ischemic heart disease [81].

There are several subclasses of flavonoids, of which the catechin class of flavanols present in cocoa and green tea have received substantial interest because of their anti-oxidant properties together with the ability to inhibit the secretion of pro-inflammatory cytokines and chemokines from activated endothelial cells [82]. This had led to the use of flavanols, particularly in the form of cocoa, in preliminary trials in order to evaluate their effectiveness in reducing CVD. A small trial involving 27 healthy individuals on a flavanol-rich diet for 5 days revealed an increase in NO production which was also associated with improved vasodilation [83]. This has subsequently been replicated in more recent trials [84,85]. End stage renal disease (ESRD) sufferers have an increased risk of CVD due to impairment of their vascular function [86]. A trial involving 57 ESRD patients found improved vascular function, determined by increased flow-mediated dilation (FMD), and reduced blood pressure following 30 days of cocoa flavanol-rich dietary supplementation [84]. Another population that has a high risk of CVD is those who are overweight. Dietary intake of 814 mg/day of flavanols for 4 weeks increased vasodilation in 30 overweight adults, however there were no changes in HDL, LDL-C or total cholesterol levels between those receiving flavanols and the control group [85]. The ability of flavanols to increase vasodilation, potentially via amplified NO production, highlights a possible mechanism by which they are capable of reducing risk of CVD. A cocoa-enriched calorie restricted diet in 50 healthy individuals has also been shown to reduce their oxLDL levels after 4 weeks when compared to calorie restriction only [87], another potential mechanism for cardiovascular protection by flavanols.

Despite the positive trials mentioned above, there have been other studies that have failed to find any improvements in blood pressure following flavanol intake [88,89]. One trial involving 32 pre- or mild-hypertensive men was unable to observe any changes in blood pressure or lipid profile following high daily intake of flavanols (1064 mg/day) for 6 weeks [88]. A lack of reduction in blood pressure, LDL-C and total cholesterol levels was also found in a study in which 24 healthy and 20 moderately hypercholesterolaemic patients received 30 g of cocoa per day for 4 weeks [89]. Nevertheless, this study did detect other cardiovascular protective effects including increased HDL levels, and a reduction in the level of the pro-inflammatory cytokine IL-1β. However, it is worth noting that approximately 34% of the cocoa treatment was made up of fiber which has also been associated with cardiovascular health benefits (discussed in Section 7 of this review), therefore the altered levels of HDL and IL-1β in this study cannot be solely associated to flavanol intake. Overall, it is difficult to draw any concrete conclusions about flavanols from these studies due to their limited participant numbers, which again highlights the need for larger clinical trials.

Recently there have been three larger studies, Cocoa, Cognition and Aging (CoCoA) [90], the Flaviola Health study [91] and PREDIMED [69], which aimed to further investigate the potential cardio-protective effects of flavanols. During the CoCoA study, 90 elderly individuals consumed either a high (993 mg), intermediate (520 mg) or low (48 mg) daily flavanol dose for 8 weeks [90]. At the end of the trial those who had received either the high or intermediate daily dose of flavanols had significant improvements in their insulin resistance, blood pressure as well as a reduction in the amount of lipid peroxidation when compared to those receiving the low flavanol dose [90]. Similar observations were found in the Flaviola Health study in which 100 healthy men and women without a history of CVD received 900 mg of cocoa flavanol every day for 1 month [91]. During the study, flavanol supplementation significantly increased FMD and HDL levels while simultaneously reducing blood pressure, arterial stiffness as well as total and LDL-C levels [91]. The largest observational study was the PREDIMED trial that involved 7172 participants whose flavanol intake ranged from 90 mg/day to 263 mg/day and they were followed for approximately 4 years [69]. The study found a significant association between increased flavanol intake and reduced CVD risk, even after controlling for other risk factors, those on lipid lowering therapies and other nutrients [69]. Despite these larger studies showing associations between flavanol intake and cardiovascular health benefits, there is still a need to perform larger trials which include individuals from multiple countries, in the presence and absence of other lipid lowering therapies in order to derive conclusive evidence for the cardiovascular protective effects of flavanols in high risk individuals.

## 7 Dietary fiber, gut microbiota, prebiotics and probiotics

Many studies have revealed cardiovascular health benefits of dietary fiber. Meta-analysis of 10 prospective cohort studies from the US and Europe involving 91,058 men and 245,186 women over 6 to 10 years follow-up demonstrated inverse correlation between consumption of dietary fiber from cereals and fruits with CHD risk [92]. A 14% reduction in risk of all coronary events and a 27% decrease in risk of coronary death were associated with each 10 g per day increase in total dietary fiber intake [92]. In addition, a study involving 46,032 men over a 12-year follow-up period found an inverse association between intake of cereal fiber (but not fruit, vegetable and total fiber) with peripheral arterial disease risk [93]. Furthermore, a study on 78,779 US women during an 18-year follow-up revealed reduced risk of total and hemorrhagic stroke with high consumption of cereal fiber [94]. However, a prospective 6-year follow-up study on 39,876 females found reduced risk of CVD and MI with a higher intake of fiber though this was not significant after adjusting for multiple compounding factors [95]. In 116 subjects under highly effective lipid-lowering therapy, fiber also had no additional effect on plasma lipid profile but produced other favorable outcomes such as reduction in body mass index and blood glucose [96].

Atherosclerosis-associated changes in metabolites and inflammatory markers produced by feeding a western diet to LDL receptor deficient mice (LDLR^−/−^) (a mouse model of the disease) is reversed in part by switching them to a fiber-rich chow diet [97]. The molecular mechanisms underlying such anti-inflammatory and -atherogenic actions of fiber are poorly understood though the gut microbiota and factors produced by them is likely to play an important role. Indeed, studies in animals administered with prebiotics (plant-derived fibers that improve the composition and function of the gut microbiota) demonstrate anti-atherogenic effects [98,99]. For example, a study in apolipoprotein E deficient mice (ApoE^−/−^), another mouse model for atherosclerosis, showed that α-cyclodextrin, which is considered as a dietary fiber, reduced the disease by modifying the gut flora [99] together with other mechanisms (e.g. increase in cholesterol solubility, cellular cholesterol efflux and anti-inflammatory responses) [100]. Fermentation of dietary fiber by gut microbiota can also generate a range of short chain fatty acids (e.g. butyrate, acetate, propionate) with anti-atherogenic properties. In support for this, butyrate reduced plaque inflammation and oxidative stress in the ApoE^−/−^ mouse model system [101,102].

Dietary manipulation of the gut microbiota is also being explored for the prevention of atherosclerosis though only limited studies have been published and mechanistic understanding remains poor. l-carnitine, a nutrient in red meat, is metabolized by the gut microbiota to trimethylamine that is then converted to the highly atherogenic trimethylamine-*N*-oxide (TMAO) by the host liver [103,104]. Interestingly, the polyphenol resveratrol remodeled the gut microbiota in mouse model systems to reduce TMAO-induced atherosclerosis and enhanced fecal bile acid excretion [105]. In addition, red wine polyphenols modulated fecal microbiota to reduce markers of metabolic syndrome in obese patients [106]. Probiotics (live bacteria with health benefits), particularly *Lactobacilli*, have also been evaluated in small human trials and animal models. For example, meta-analysis of 15 studies with 788 subjects showed effective lowering of cardiovascular risk factors such as total cholesterol, LDL-C and inflammatory markers [107], conclusions that are supported by another similar meta-analysis [108]. Studies in mouse model systems have also shown attenuation of atherosclerosis by probiotic bacteria via multiple mechanisms, including induction of tolerogenic dendritic cells [109], stimulation of cholesterol efflux [110] and reduction in both intestinal cholesterol uptake and vascular inflammation [111,112].

## 8 Other nutraceuticals

There are several other nutraceuticals that have been less well studied so will only be mentioned briefly here with focus on outcomes from animal models and clinical trials (Table 2). Allicin, the primary active ingredient in garlic, attenuated atherosclerosis in both the LDLR^−/−^ and ApoE^−/−^ model systems by acting as an antioxidant, and inhibiting LDL uptake and degradation by macrophages [113]. However, meta-analysis of 45 trials showed that garlic supplementation produced only short-term (1–3 months) reduction in plasma levels of total cholesterol, LDL-C and TG with no effect on blood pressure or glucose levels [114]. Berberine, a plant alkaloid, also reduced plaque size and vulnerability, inflammation and oxidative stress in ApoE^−/−^ mice [115,116]. Clinical trials have been small (63–144 patients) but demonstrated reduced plasma levels of total cholesterol and LDL-C via a mechanism involving increased LDLR mRNA stability [[117], [118], [119]]. However, the bioavailability of berberine is very poor (<1%) [120]. Although carnosine, an antioxidant peptide commonly found in meat, attenuated atherosclerosis in ApoE^−/−^ mice by lowering levels of TG and reducing vascular inflammation and foam cell formation [[121], [122], [123]], a small pilot study in 15 humans found no changes in blood pressure or serum levels of cholesterol and CRP [124]. For lycopene, a carotenoid found in tomatoes and other red fruit and vegetables, two systematic reviews and meta-analysis published in 2017 showed that higher intake is associated with a lower risk of CVD due to reduction in LDL-C, pro-inflammatory cytokines and blood pressure, and improved FMD [125,126]. Coenzyme Q_10_ (CoQ_10_), an anti-oxidant and component of the electron transport chain whose de novo synthesis is reduced by statin therapy, attenuated atherosclerosis in the ApoE^−/−^ model system by promoting macrophage cholesterol efflux [127], which was also confirmed in a pilot study on 20 healthy volunteers [128]. A meta-analysis involving 194 patients also demonstrated significant improvement of endothelial function with CoQ_10_ supplementation [129].

Phytosterols, plant sterols and stanols, are believed to mediate cardio-protective actions by competing with cholesterol in the intestinal lumen during dietary and biliary cholesterol uptake. For phytosterols, an epidemiological study involving 22,256 individuals showed correlations between diets with high levels and reduced plasma LDL-C concentration [130]. A meta-analysis of 41 trials showed about 10% lowering of LDL-C by intake of 2 g per day of phytosterols with effects being additive with intervention by drugs or diet [131]. For example, a 20% reduction in plasma LDL-C was found by diets high in phytosterols and low in saturated fats and cholesterol [131]. Adding phytosterols to statins was also found to be more effective than doubling the dose of the latter [131]. Although this study failed to see any additional reduction in LDL-C with intakes of phytosterols above 2 g per day, this has been observed in other studies [132,133]. More recently, meta-analysis involving 1308 subjects showed an association between phytosterol intake and reduced plasma LDL-C levels but no changes in plasma CRP [134]. On the basis of such promising outcomes, several guidelines recommend daily intake of 2 g of plant sterols and/or stanols to reduce LDL-C levels [135]. Foods enriched with phytosterols (e.g. margarine) are often taken to achieve these recommended levels because western diets typically contain about 300 mg/day of phytosterols [135]. Phytosterols also attenuate atherosclerosis in mouse model systems by reducing inflammation and plasma cholesterol levels, and increasing HDL [136,137]. However, this has not been replicated in humans with some concerns raised on the beneficial effects of food supplementation with phytosterols (see [135,138,139] for recent reviews), emphasizing the need for further research and large trials.

## 9 Conclusions

Nutraceuticals represent promising agents in the prevention and treatment of atherosclerosis as demonstrated by both preclinical and clinical studies. More research is essential given that current therapies are not fully effective and many promising pharmaceutical leads have failed at the clinical level. However, research on nutraceuticals have lagged considerably behind pharmaceuticals on several aspects: (i) large, controlled clinical trials on a scale at least similar to the REDUCE-IT and STRENGTH trials currently under way for omega-3 PUFA supplementation and possibly on the scale of major pharmaceuticals such as ezetimibe and PCSK9 antibodies; (ii) pre-clinical studies that use concentrations within the physiological level (most use very high doses), could be easily translated to humans (many are carried out on mice that show differences to humans in two key aspects of CVD, lipid metabolism and the inflammatory response) and also incorporate dose-response experiments whenever possible. In addition, it is essential that there is consistency in human studies in relation to composition, dose of agent and duration of intervention in order to reduce the significant level of discrepancy that often exists in published literature on nutraceutical actions and efficacy. Deeper insight into the mechanisms underlying nutraceutical actions is also required as they could inform on the development of potent pharmacological agents that mimic key responses. For example, studies on omega-3 fatty acids revealed that GPR120 was the receptor that mediated its potent anti-inflammatory and insulin sensitizing effects [140]. Further research demonstrated that a selective high-affinity, orally available, small molecule GPR120 agonist (cpdA) improved insulin resistance and chronic inflammation in obese mice [141]. We are indeed entering an exciting phase in nutraceutical research.

## Transparency document

Transparency document. Image 1

## References

[1] Benjamin E.J., Blaha M.J., Chiuve S.E. (2017). Heart disease and stroke statistics-2017 update: a report from the American Heart Association. Circulation.

[2] Buckley M.L., Ramji D.P. (2015). The influence of dysfunctional signaling and lipid homeostasis in mediating the inflammatory responses during atherosclerosis. Biochim. Biophys. Acta Mol. Basis Dis..

[3] McLaren J.E., Michael D.R., Ashlin T.G., Ramji D.P. (2011). Cytokines, macrophage lipid metabolism and foam cells: implications for cardiovascluar disease therapy. Prog. Lipid Res..

[4] Moss J.W.E., Ramji D.P. (2016). Cytokines: roles in atherosclerosis disease progression and potential therapeutic targets. Future Med. Chem..

[5] Salter R.C., Foka P., Davies T.S., Gallagher H., Michael D.R., Ashlin T.G., Ramji D.P. (2016). The role of mitogen-activated protein kinases and sterol receptor coactivator-1 in TGF-β-regulated expression of genes implicated in macrophage cholesterol uptake. Sci. Rep..

[6] Shapiro M.D., Fazio S. (2016). From lipids to inflammation: new approaches to reducing atherosclerotic risk. Circ. Res..

[7] Ramkumar S., Raghunath A., Raghunath S. (2016). Statin therapy: review of safety and potential side effects. Acta Cardiol. Sin..

[8] Murphy S.A., Cannon C.P., Blazing M.A. (2016). Reduction in total cardiovascular events with ezetimibe/simvastatin post-acute coronary syndrome: the IMPROVE-IT trial. J. Am. Coll. Cardiol..

[9] Tsujita K., Sugiyama S., Sumida H. (2015). Impact of dual lipid-lowering strategy with ezetimibe and atorvastatin on coronary plaque regression in patients with percutaneous coronary intervention, the multicenter randomized controlled PRECISE-IVUS trial. J. Am. Coll. Cardiol..

[10] Kim K.J., Kim S.H., Yoon Y.W. (2016). Effect of fixed-dose combinations of ezetimibe plus rosuvastatin in patients with primary hypercholesterolemia: MRS-ROZE (Multicenter Randomized Study of ROsuvastatin and eZEtimibe). Cardiovasc. Ther..

[11] Preiss D., Mafham M. (2017). PCSK9 inhibition: the dawn of a new age in cholesterol lowering?. Diabetologia.

[12] Sabatine M.S., Giugliano R.P., Keech A.C. (2017). Evolocumab and clinical outcomes in patients with cardiovascular disease. N. Engl. J. Med..

[13] Ridker P.M., Tardif J.-C., Amarenco P. (2017). Lipid-reduction variability and antidrug-antibody formation with bococizumab. N. Engl. J. Med..

[14] Ridker P.M., Revkin J., Amarenco P. (2017). Cardiovascular efficacy and safety of bococizumab in high-risk patients. N. Engl. J. Med..

[15] Ray K.K., Landmesser U., Leiter L.A. (2017). Inclisiran in patients at high cardiovascular risk with elevated LDL cholesterol. N. Engl. J. Med..

[16] Ladeiras-Lopes R., Agewall S., Tawakol A. (2015). Atherosclerosis: recent trials, new targets and future directions. Int. J. Cardiol..

[17] Kingwell B.A., Chapman M.J., Kontush A., Miller N.E. (2014). HDL-targeted therapies: progress, failures and future. Nat. Rev. Drug Discov..

[18] Ridker P.M., Everett B.M., Thuren T. (2017). Antiinflammatory therapy with canakinumab for atherosclerotic disease. N. Engl. J. Med..

[19] Ravera A., Carubelli V., Sciatti E. (2016). Nutrition and cardiovascular disease: finding the perfect recipe for cardiovascular health. Nutrients.

[20] Ozkanlar S., Akcay F. (2012). Antioxidant vitamins in atherosclerosis—animal experiments and clinical studies. Adv. Clin. Exp. Med..

[21] Sesso H.D., Buring J.E., Christen W.G. (2008). Vitamins E and C in the prevention of cardiovascular disease in men. JAMA.

[22] Zureik M., Galan P., Bertrais S. (2004). Effects of long-term daily low-dose supplementation with antioxidant vitamins and minerals on structure and function of large arteries. Arterioscler. Thromb. Vasc. Biol..

[23] Hodis H.N., Mack W.J., LaBree L. (2002). Alpha-tocopherol supplementation in healthy individuals reduces low-density lipoprotein oxidation but not atherosclerosis: the Vitamin E Atherosclerosis Prevention Study (VEAPS). Circulation.

[24] Heart Protection Study Collaborative Group (2002). MRC/BHF Heart Protection Study of antioxidant vitamin supplementation in 20,536 high-risk individuals: a randomised placebo-controlled trial. Lancet.

[25] Anon (1999). Dietary supplementation with n-3 polyunsaturated fatty acids and vitamin E after myocardial infarction: results of the GISSI-Prevenzione trial. Lancet.

[26] Salonen J.T., Nyyssönen K., Salonen R. (2000). Antioxidant Supplementation in Atherosclerosis Prevention (ASAP) study: a randomized trial of the effect of vitamins E and C on 3-year progression of carotid atherosclerosis. J. Intern. Med..

[27] Lee I.-M., Cook N.R., Gaziano J.M. (2005). Vitamin E in the primary prevention of cardiovascular disease and cancer. JAMA.

[28] Karppi J., Kurl S., Laukkanen J.A., Rissanen T.H., Kauhanen J. (2011). Plasma carotenoids are related to intima-media thickness of the carotid artery wall in men from eastern Finland. J. Intern. Med..

[29] Karppi J., Kurl S., Ronkainen K., Kauhanen J., Laukkanen J.A. (2013). Serum carotenoids reduce progression of early atherosclerosis in the carotid artery wall among Eastern Finnish men. PLoS One.

[30] Calder P.C. (2012). Omega-3 polyunsaturated fatty acids and inflammatory processes: nutrition or pharmacology?. Br. J. Clin. Pharmacol..

[31] Nakajima K., Yamashita T., Kita T. (2011). Orally administered eicosapentaenoic acid induces rapid regression of atherosclerosis via modulating the phenotype of dendritic cells in LDL receptor-deficient mice. Arterioscler. Thromb. Vasc. Biol..

[32] Brown A.L., Zhu X., Rong S. (2012). Omega-3 fatty acids ameliorate atherosclerosis by favorably altering monocyte subsets and limiting monocyte recruitment to aortic lesions. Arterioscler. Thromb. Vasc. Biol..

[33] Yokoyama M., Origasa H., Matsuzaki M. (2007). Effects of eicosapentaenoic acid on major coronary events in hypercholesterolaemic patients (JELIS): a randomised open-label, blinded endpoint analysis. Lancet.

[34] Bhatt D.L., Steg P.G., Brinton E.A. (2017). Rationale and design of REDUCE-IT: reduction of cardiovascular events with icosapent ethyl-intervention trial. Clin. Cardiol..

[35] Franzese C.J., Bliden K.P., Gesheff M.G. (2015). Relation of fish oil supplementation to markers of atherothrombotic risk in patients with cardiovascular disease not receiving lipid-lowering therapy. Am. J. Cardiol..

[36] Rauch B., Schiele R., Schneider S. (2010). OMEGA, a randomized, placebo-controlled trial to test the effect of highly purified omega-3 fatty acids on top of modern guideline-adjusted therapy after myocardial infarction. Circulation.

[37] The ORIGIN Trial Investigators (2012). n–3 fatty acids and cardiovascular outcomes in patients with dysglycemia. N. Engl. J. Med..

[38] Bays H.E., Ballantyne C.M., Kastelein J.J., Isaacsohn J.L., Braeckman R.A., Soni P.N. (2011). Eicosapentaenoic acid ethyl ester (AMR101) therapy in patients with very high triglyceride levels (from the Multi-center, plAcebo-controlled, Randomized, double-blINd, 12-week study with an open-label Extension [MARINE] trial). Am. J. Cardiol..

[39] Ballantyne C.M., Bays H.E., Kastelein J.J. (2012). Efficacy and safety of eicosapentaenoic acid ethyl ester (AMR101) therapy in statin-treated patients with persistent high triglycerides (from the ANCHOR study). Am. J. Cardiol..

[40] Bays H.E., Ballantyne C.M., Braeckman R.A., Stirtan W.G., Soni P.N. (2013). Icosapent ethyl, a pure ethyl ester of eicosapentaenoic acid: effects on circulating markers of inflammation from the MARINE and ANCHOR studies. Am. J. Cardiovasc. Drugs.

[41] U.S. National Institutes of Health (2017). A study of AMR101 to evaluate its ability to reduce cardiovascular events in high risk patients with hypertriglyceridemia and on statin. https://clinicaltrials.gov/ct2/show/NCT01492361.

[42] U.S. National Institutes of Health (2017). Outcomes study to assess STatin Residual Risk Reduction With EpaNova in HiGh CV Risk PatienTs With Hypertriglyceridemia (STRENGTH). https://clinicaltrials.gov/ct2/show/NCT02104817.

[43] Moss J.W.E., Ramji D.P. (2016). Nutraceutical therapies for atherosclerosis. Nat. Rev. Cardiol..

[44] American Heart Association (2016). Fish and omega-3 fatty acids. Am. Heart Assoc..

[45] Wang X., Lin H., Gu Y. (2012). Multiple roles of dihomo-γ-linolenic acid against proliferation diseases. Lipids Health Dis..

[46] Fan Y.Y., Ramos K.S., Chapkin R.S. (2001). Dietary gamma-linolenic acid suppresses aortic smooth muscle cell proliferation and modifies atherosclerotic lesions in apolipoprotein E knockout mice. J. Nutr..

[47] Takai S., Jin D., Kawashima H. (2009). Anti-atherosclerotic effects of dihomo-γ-linolenic acid in ApoE-deficient mice. J. Atheroscler. Thromb..

[48] Al-Khudairy L., Hartley L., Clar C., Flowers N., Hooper L., Rees K. (2015). Omega 6 fatty acids for the primary prevention of cardiovascular disease. Cochrane Database Syst. Rev..

[49] Katan M.B. (2009). Omega-6 polyunsaturated fatty acids and coronary heart disease. Am. J. Clin. Nutr..

[50] Oh K., Hu F.B., Manson J.E., Stampfer M.J., Willett W.C. (2005). Dietary fat intake and risk of coronary heart disease in women: 20 years of follow-up of the Nurses' Health Study. Am. J. Epidemiol..

[51] Laaksonen D.E., Nyyssönen K., Niskanen L., Rissanen T.H., Salonen J.T. (2005). Prediction of cardiovascular mortality in middle-aged men by dietary and serum linoleic and polyunsaturated fatty acids. Arch. Intern. Med..

[52] Guivernau M., Meza N., Barja P., Roman O. (1994). Clinical and experimental study on the long-term effect of dietary gamma-linolenic acid on plasma lipids, platelet aggregation, thromboxane formation, and prostacyclin production. Prostaglandins Leukot. Essent. Fat. Acids.

[53] Tomiyama H., Matsumoto C., Odaira M. (2011). Relationships among the serum omega fatty acid levels, serum C-reactive protein levels and arterial stiffness/wave reflection in Japanese men. Atherosclerosis.

[54] Griffin M.D., Sanders T.A.B., Davies I.G. (2006). Effects of altering the ratio of dietary n-6 to n-3 fatty acids on insulin sensitivity, lipoprotein size, and postprandial lipemia in men and postmenopausal women aged 45–70 y: the OPTILIP study. Am. J. Clin. Nutr..

[55] Bahramsoltani R., Ebrahimi F., Farzaei M.H. (2017). Dietary polyphenols for atherosclerosis: a comprehensive review and future perspectives. Crit. Rev. Food Sci. Nutr..

[56] Granados-Principal S., Quiles J.L., Ramirez-Tortosa C.L., Sanchez-Rovira P., Ramirez-Tortosa M.C. (2010). Hydroxytyrosol: from laboratory investigations to future clinical trials. Nutr. Rev..

[57] Xu J., Zhou X., Deng Q., Huang Q., Yang J., Huang F. (2011). Rapeseed oil fortified with micronutrients reduces atherosclerosis risk factors in rats fed a high-fat diet. Lipids Health Dis..

[58] Wongwarawipat T., Papageorgiou N., Bertsias D., Siasos G., Tousoulis D. (2018). Olive oil-related anti-inflammatory effects on atherosclerosis: potential clinical implications. Endocr Metab Immune Disord Drug Targets.

[59] Attori L., Di Biase A., Di Benedetto R., Rogato P., Di Virgilio A., Salvati S. (2010). Micronutrient-enriched rapeseed oils reduce cardiovascular disease risk factors in rats fed a high-fat diet. Atherosclerosis.

[60] Tjelle T.E., Holtung L., Bøhn S.K. (2015). Polyphenol-rich juices reduce blood pressure measures in a randomised controlled trial in high normal and hypertensive volunteers. Br. J. Nutr..

[61] Shema-Didi L., Kristal B., Sela S., Geron R., Ore L. (2014). Does pomegranate intake attenuate cardiovascular risk factors in hemodialysis patients?. Nutr. J..

[62] Ras R.T., Zock P.L., Zebregs Y.E.M.P., Johnston N.R., Webb D.J., Draijer R. (2013). Effect of polyphenol-rich grape seed extract on ambulatory blood pressure in subjects with pre- and stage I hypertension. Br. J. Nutr..

[63] Bondia-Pons I., Pöhö P., Bozzetto L. (2014). Isoenergetic diets differing in their n-3 fatty acid and polyphenol content reflect different plasma and HDL-fraction lipidomic profiles in subjects at high cardiovascular risk. Mol. Nutr. Food Res..

[64] Covas M.I., Nyyssonen K., Poulsen H.E. (2006). The effect of polyphenols in olive oil on heart disease risk factors: a randomized trial. Ann. Intern. Med..

[65] Panahi Y., Hosseini M.S., Khalili N., Naimi E., Majeed M., Sahebkar A. (2015). Antioxidant and anti-inflammatory effects of curcuminoid-piperine combination in subjects with metabolic syndrome: a randomized controlled trial and an updated meta-analysis. Clin. Nutr..

[66] Sahebkar A. (2014). A systematic review and meta-analysis of randomized controlled trials investigating the effects of curcumin on blood lipid levels. Clin. Nutr..

[67] Sahebkar A. (2014). Are curcuminoids effective C-reactive protein-lowering agents in clinical practice? Evidence from a meta-analysis. Phytother. Res..

[68] Shin S.K., Ha T.Y., McGregor R.A., Choi M.S. (2011). Long-term curcumin administration protects against atherosclerosis via hepatic regulation of lipoprotein cholesterol metabolism. Mol. Nutr. Food Res..

[69] Tresserra-Rimbau A., Rimm E.B., Medina-Remón A. (2014). Inverse association between habitual polyphenol intake and incidence of cardiovascular events in the PREDIMED study. Nutr. Metab. Cardiovasc. Dis..

[70] Tresserra-Rimbau A., Rimm E.B., Medina-Remón A. (2014). Polyphenol intake and mortality risk: a re-analysis of the PREDIMED trial. BMC Med..

[71] Medina-Remón A., Tresserra-Rimbau A., Pons A. (2015). Effects of total dietary polyphenols on plasma nitric oxide and blood pressure in a high cardiovascular risk cohort. The PREDIMED randomized trial. Nutr. Metab. Cardiovasc. Dis..

[72] Guo X., Tresserra-Rimbau A., Estruch R. (2016). Effects of polyphenol, measured by a biomarker of total polyphenols in urine, on cardiovascular risk factors after a long-term follow-up in the PREDIMED study. Oxidative Med. Cell. Longev..

[73] Medina-Remón A., Casas R., Tressserra-Rimbau A. (2017). Polyphenol intake from a Mediterranean diet decreases inflammatory biomarkers related to atherosclerosis: a substudy of the PREDIMED trial. Br. J. Clin. Pharmacol..

[74] Mulvihill E.E., Burke A.C., Huff M.W. (2016). Citrus flavonoids as regulators of lipoprotein metabolism and atherosclerosis. Annu. Rev. Nutr..

[75] Fuhrman B., Aviram M. (2001). Flavonoids protect LDL from oxidation and attenuate atherosclerosis. Curr. Opin. Lipidol..

[76] Aviram M., Fuhrman B. (2002). Wine flavonoids protect against LDL oxidation and atherosclerosis. Ann. N. Y. Acad. Sci..

[77] Aviram M., Dornfeld L., Kaplan M. (2002). Pomegranate juice flavonoids inhibit low-density lipoprotein oxidation and cardiovascular diseases: studies in atherosclerotic mice and in humans. Drugs Exp. Clin. Res..

[78] Faggio C., Sureda A., Morabito S. (2017). Flavonoids and platelet aggregation: a brief review. Eur. J. Pharmacol..

[79] Hertog M.G., Feskens E.J., Hollman P.C., Katan M.B., Kromhout D. (1993). Dietary antioxidant flavonoids and risk of coronary heart disease: the Zutphen Elderly Study. Lancet.

[80] Hertog M.G., Feskens E.J., Kromhout D. (1997). Antioxidant flavonols and coronary heart disease risk. Lancet.

[81] Hertog M.G., Sweetnam P.M., Fehily A.M., Elwood P.C., Kromhout D. (1997). Antioxidant flavonols and ischemic heart disease in a Welsh population of men. The Caerphilly Study. Am. J. Clin. Nutr..

[82] Yamakuchi M., Bao C., Ferlito M., Lowenstein C.J. (2008). Epigallocatechin gallate inhibits endothelial exocytosis. Biol. Chem..

[83] Fisher N.D., Hughes M., Gerhard-Herman M., Hollenberg N.K. (2003). Flavanol-rich cocoa induces nitric-oxide-dependent vasodilation in healthy humans. J. Hypertens..

[84] Rassaf T., Rammos C., Hendgen-Cotta U.B. (2016). Vasculoprotective effects of dietary cocoa flavanols in patients on hemodialysis: a double-blind, randomized, placebo-controlled trial. Clin. J. Am. Soc. Nephrol..

[85] West S.G., McIntyre M.D., Piotrowski M.J. (2014). Effects of dark chocolate and cocoa consumption on endothelial function and arterial stiffness in overweight adults. Br. J. Nutr..

[86] Zimmerli L.U., Mark P.B., Steedman T. (2007). Vascular function in patients with end-stage renal disease and/or coronary artery disease: a cardiac magnetic resonance imaging study. Kidney Int..

[87] Ibero-Baraibar I., Abete I., Navas-Carretero S., Massis-Zaid A., Martinez J.A., Zulet M.A. (2014). Oxidised LDL levels decreases after the consumption of ready-to-eat meals supplemented with cocoa extract within a hypocaloric diet. Nutr. Metab. Cardiovasc. Dis..

[88] Rull G., Mohd-Zain Z.N., Shiel J. (2015). Effects of high flavanol dark chocolate on cardiovascular function and platelet aggregation. Vasc. Pharmacol..

[89] Sarriá B., Martínez-López S., Sierra-Cinos J.L., García-Diz L., Mateos R., Bravo L. (2014). Regular consumption of a cocoa product improves the cardiometabolic profile in healthy and moderately hypercholesterolaemic adults. Br. J. Nutr..

[90] Mastroiacovo D., Kwik-Uribe C., Grassi D. (2015). Cocoa flavanol consumption improves cognitive function, blood pressure control, and metabolic profile in elderly subjects: the Cocoa, Cognition, and Aging (CoCoA) Study—a randomized controlled trial. Am. J. Clin. Nutr..

[91] Sansone R., Rodriguez-Mateos A., Heuel J. (2015). Cocoa flavanol intake improves endothelial function and Framingham Risk Score in healthy men and women: a randomised, controlled, double-masked trial: the Flaviola Health Study. Br. J. Nutr..

[92] Pereira M.A., O'Reilly E., Augustsson K. (2004). Dietary fiber and risk of coronary heart disease: a pooled analysis of cohort studies. Arch. Intern. Med..

[93] Merchant A.T., Hu F.B., Spiegelman D., Willett W.C., Rimm E.B., Ascherio A. (2003). Dietary fiber reduces peripheral arterial disease risk in men. J. Nutr..

[94] Oh K., Hu F.B., Cho E. (2005). Carbohydrate intake, glycemic index, glycemic load, and dietary fiber in relation to risk of stroke in women. Am. J. Epidemiol..

[95] Liu S.M., Buring J.E., Sesso H.D., Rimm E.B., Willett W.C., Manson J.E. (2002). A prospective study of dietary fiber intake and risk of cardiovascular disease among women. J. Am. Coll. Cardiol..

[96] Ramos S.C., Fonseca F.A., Kasmas S.H. (2011). The role of soluble fiber intake in patients under highly effective lipid-lowering therapy. Nutr. J..

[97] Li D., Zhang L., Dong F. (2015). Metabonomic changes associated with atherosclerosis progression for LDLR(−/−) mice. J. Proteome Res..

[98] Rault-Nania M.H., Gueux E., Demougeot C., Demigné C., Rock E., Mazur A. (2006). Inulin attenuates atherosclerosis in apolipoprotein E-deficient mice. Br. J. Nutr..

[99] Sakurai T., Sakurai A., Chen T. (2017). Dietary α-cyclodextrin reduces atherosclerosis and modifies gut flora in apolipoprotein E-deficient mice. Mol. Nutr. Food Res..

[100] Zimmer S., Grebe A., Bakke S.S. (2016). Cyclodextrin promotes atherosclerosis regression via macrophage reprogramming. Sci. Transl. Med..

[101] Aguilar E.C., Leonel A.J., Teixeira L.G. (2014). Butyrate impairs atherogenesis by reducing plaque inflammation and vulnerability and decreasing NFκB activation. Nutr. Metab. Cardiovasc. Dis..

[102] Aguilar E.C., Santos L.C., Leonel A.J. (2016). Oral butyrate reduces oxidative stress in atherosclerotic lesion sites by a mechanism involving NADPH oxidase down-regulation in endothelial cells. J. Nutr. Biochem..

[103] Koeth R.A., Wang Z., Levison B.S. (2013). Intestinal microbiota metabolism of l-carnitine, a nutrient in red meat, promotes atherosclerosis. Nat. Med..

[104] Koeth R.A., Levison B.S., Culley M.K. (2014). γ-Butyrobetaine is a proatherogenic intermediate in gut microbial metabolism of l-carnitine to TMAO. Cell Metab..

[105] Chen M.L., Yi L., Zhang Y. (2016). Resveratrol attenuates trimethylamine-N-oxide (TMAO)-induced atherosclerosis by regulating TMAO synthesis and bile acid metabolism via remodeling of the gut microbiota. MBio.

[106] Moreno-Indias I., Sánchez-Alcoholado L., Pérez-Martínez P. (2016). Red wine polyphenols modulate fecal microbiota and reduce markers of the metabolic syndrome in obese patients. Food Funct..

[107] Sun J., Buys N. (2015). Effects of probiotics consumption on lowering lipids and CVD risk factors: a systematic review and meta-analysis of randomized controlled trials. Ann. Med..

[108] Shimizu M., Hashiguchi M., Shiga T., Tamura H.O., Mochizuki M. (2015). Meta-analysis: effects of probiotic supplementation on lipid profiles in normal to mildly hypercholesterolemic individuals. PLoS ONE.

[109] Mizoguchi T., Kasahara K., Yamashita T. (2017). Oral administration of the lactic acid bacterium *Pediococcus acidilactici* attenuates atherosclerosis in mice by inducing tolerogenic dendritic cells. Heart Vessel..

[110] Hong Y.F., Kim H., Kim H.S., Park W.J., Kim J.Y., Chung D.K. (2016). *Lactobacillus acidophilus* K301 inhibits atherogenesis via induction of 24 (S), 25-epoxycholesterol-mediated ABCA1 and ABCG1 production and cholesterol efflux in macrophages. PLoS One.

[111] Huang Y., Wang J., Quan G., Wang X., Yang L., Zhong L. (2014). *Lactobacillus acidophilus* ATCC 4356 prevents atherosclerosis via inhibition of intestinal cholesterol absorption in apolipoprotein E-knockout mice. Appl. Env. Microbiol..

[112] Chan Y.K., El-Nezami H., Chen Y., Kinnunen K., Kirjavainen P.V. (2016). Probiotic mixture VSL#3 reduce high fat diet induced vascular inflammation and atherosclerosis in ApoE(−/−) mice. AMB Express.

[113] Gonen A., Harats D., Rabinkov A. (2005). The antiatherogenic effect of allicin: possible mode of action. Pathobiology.

[114] Ackermann R.T., Mulrow C.D., Ramirez G., Gardner C.D., Morbidoni L., Lawrence V.A. (2001). Garlic shows promise for improving some cardiovascular risk factors. Arch. Intern. Med..

[115] Chen J., Cao J., Fang L. (2014). Berberine derivatives reduce atherosclerotic plaque size and vulnerability in apoE(−/−) mice. J. Transl. Med..

[116] Li H., He C., Wang J. (2016). Berberine activates peroxisome proliferator-activated receptor gamma to increase atherosclerotic plaque stability in Apoe(−/−) mice with hyperhomocysteinemia. J. Diabetes Investig..

[117] Kong W.-J., Wei J., Zuo Z.-Y. (2008). Combination of simvastatin with berberine improves the lipid-lowering efficacy. Metabolism.

[118] Derosa G., D'Angelo A., Bonaventura A., Bianchi L., Romano D., Maffioli P. (2013). Effects of berberine on lipid profile in subjects with low cardiovascular risk. Expert. Opin. Biol. Ther..

[119] Kong W., Wei J., Abidi P. (2004). Berberine is a novel cholesterol-lowering drug working through a unique mechanism distinct from statins. Nat. Med..

[120] Liu C.S., Zheng Y.R., Zhang Y.F., Long X.Y. (2016). Research progress on berberine with a special focus on its oral bioavailability. Fitoterapia.

[121] Menini S., Iacobini C., Ricci C. (2012). d-Carnosine octylester attenuates atherosclerosis and renal disease in ApoE null mice fed a Western diet through reduction of carbonyl stress and inflammation. Br. J. Pharmacol..

[122] Barski O.A., Xie Z., Baba S.P. (2013). Dietary carnosine prevents early atherosclerotic lesion formation in apolipoprotein E-null mice. Arterioscler. Thromb. Vasc. Biol..

[123] Brown B.E., Kim C.H.J., Torpy F.R. (2014). Supplementation with carnosine decreases plasma triglycerides and modulates atherosclerotic plaque composition in diabetic apoE−/− mice. Atherosclerosis.

[124] de Courten B., Jakubova M., de Courten M.P. (2016). Effects of carnosine supplementation on glucose metabolism: pilot clinical trial. Obesity.

[125] Song B., Liu K., Gao Y. (2017). Lycopene and risk of cardiovascular diseases: a meta-analysis of observational studies. Mol. Nutr. Food Res..

[126] Cheng H.M., Koutsidis G., Lodge J.K., Ashor A., Siervo M., Lara J. (2017). Tomato and lycopene supplementation and cardiovascular risk factors: a systematic review and meta-analysis. Atherosclerosis.

[127] Wang D., Yan X., Xia M. (2014). Coenzyme Q10 promotes macrophage cholesterol efflux by regulation of the activator protein-1/miR-378/ATP-binding cassette transporter G1–signaling pathway. Arterioscler. Thromb. Vasc. Biol..

[128] Yan X., Shen T., Jiang X. (2015). Coenzyme Q10 consumption promotes ABCG1-mediated macrophage cholesterol efflux: a randomized, double-blind, placebo-controlled, cross-over study in healthy volunteers. Mol. Nutr. Food Res..

[129] Gao L., Mao Q., Cao J., Wang Y., Zhou X., Fan L. (2012). Effects of coenzyme Q10 on vascular endothelial function in humans: a meta-analysis of randomized controlled trials. Atherosclerosis.

[130] Andersson S.W., Skinner J., Ellegård L. (2004). Intake of dietary plant sterols is inversely related to serum cholesterol concentration in men and women in the EPIC Norfolk population: a cross-sectional study. Eur. J. Clin. Nutr..

[131] Katan M.B., Grundy S.M., Jones P. (2003). Efficacy and safety of plant stanols and sterols in the management of blood cholesterol levels. Mayo Clin. Proc..

[132] Demonty I., Ras R.T., van der Knaap H.C. (2009). Continuous dose-response relationship of the LDL-cholesterol-lowering effect of phytosterol uptake. J. Nutr..

[133] Musa-Veloso K., Poon T.H., Elliot J.A., Chung C. (2011). A comparison of the LDL-cholesterol lowering efficacy of plant stanols and plant sterols over a continuous dose range: results of a meta-analysis of randomized, placebo-controlled trials. Prostaglandins Leukot. Essent. Fat. Acids.

[134] Rocha V.Z., Ras R.T., Gagliardi A.C., Mangili L.C., Trautwein E.A., Santos R.D. (2016). Effects of phytosterols on markers of inflammation: a systematic review and meta-analysis. Atherosclerosis.

[135] Cabral C.E., Klein M.R.S.T. (2017). Phytosterols in the treatment of hypercholesterolemia and prevention of cardiovascular disease. Arq. Bras. Cardiol..

[136] Nashed B., Yeganeh B., HayGlass K.T., Moghadasian M.H. (2005). Antiatherogenic effects of dietary plant sterols are associated with inhibition of proinflammatory cytokine production in Apo E-KO mice. J. Nutr..

[137] Bombo R.P., Afonso M.S., Machado R.M. (2013). Dietary phytosterol does not accumulate in the arterial wall and prevents atherosclerosis of LDLr-KO mice. Atherosclerosis.

[138] Rysz J., Franczyk B., Olszewski R., Banach M., Gluba-Brzózka A. (2017). The use of plant sterols and stanols as lipid-lowering agents in cardiovascular disease. Curr. Pharm. Des..

[139] Köhler J., Teupser D., Elsässer A., Weingärtner O. (2017). Plant sterol enriched functional food and atherosclerosis. Br. J. Pharmacol..

[140] Oh D., Talukdar S., Bae E. (2010). GPR120 is an omega-3 fatty acid receptor mediating potent anti-inflammatory and insulin-sensitizing effects. Cell.

[141] Oh D., Walenta E., Akiyama T. (2014). A Gpr120-selective agonist improves insulin resistance and chronic inflammation in obese mice. Nat. Med..

